# DEVELOPING RURAL INSIGHTS FOR BUILDING SUSTAINABLE AGE-FRIENDLY COMMUNITIES INITIATIVES

**DOI:** 10.1093/geroni/igz038.3482

**Published:** 2019-11-08

**Authors:** Elizabeth McCrillis, Mark Skinner, Amber Colibaba

**Affiliations:** 1 Trent University, Ontario, Canada; 2 Trent University, Peterborough, Ontario, Canada

## Abstract

Researchers have questioned the focus on describing features of preliminary age-friendly implementation and the absence of program evaluations or analyses of long-term implementation. This lack of knowledge inadvertently simplifies unique pathways to age-friendly sustainability, preventing researchers from conducting in-depth, retrospective examinations of age-friendly and post-age-friendly perspectives. Seeking to address this critique, this research examines the challenges to rural age-friendly program sustainability, and the factors that may help committees overcome these barriers. Data were collected through a succession of qualitative studies, including a provincial age-friendly program evaluation and a series of studies examining sustainability in rural initiatives. Eighty in-depth interviews with age-friendly leaders and older participants from 27 rural Canadian programs were conducted, seeking knowledge about programs’ development and implementation. Key findings include the conceptualization of an implementation gap between early development and long-term viability, the important role played by individual communities, the challenges of capacity and jurisdictional fragmentation, and the inability of rural age-friendly programs to tackle bigger picture issues such as housing and transportation given their necessarily limited scope and reach. Implications relevant for research and practice suggest that drawing on individual, community, and jurisdictional factors will maximize the success and sustainability of rural age-friendly programs, thereby extending the reach and scale of programs to more directly affect older people. From this, we conclude that the sustainability and success of rural age-friendly programs would benefit from consistent, renewable government funding that considers factors relevant to overcoming the implementation gap and challenges created by jurisdictional fragmentation and de-emphasizing community individuality.

